# Persistent foetal vasculature arising from the peripheral retina

**DOI:** 10.1093/omcr/omag054

**Published:** 2026-05-10

**Authors:** Panagiotis Tsoutsanis, Piergiacomo Grassi

**Affiliations:** Department of Ophthalmology, Northern Care Alliance NHS Foundation Trust, Rochdale Infirmary, Whitehall St, Rochdale OL12 0NB, United Kingdom; School of Medical Sciences, Faculty of Biology, Medicine and Health, The University of Manchester, Stopford Building, Oxford Road, Manchester M13 9PT, United Kingdom; Department of Ophthalmology, Northern Care Alliance NHS Foundation Trust, Rochdale Infirmary, Whitehall St, Rochdale OL12 0NB, United Kingdom; School of Medical Sciences, Faculty of Biology, Medicine and Health, The University of Manchester, Stopford Building, Oxford Road, Manchester M13 9PT, United Kingdom

**Keywords:** medical ophthalmology

A 19-year-old female patient presented with a 2-month history of daily episodes of right eye photopsia. No past medical history other than myopia was reported and she was born in term. Visual acuity was 6/6 with glasses bilaterally.

**Figure 1:**
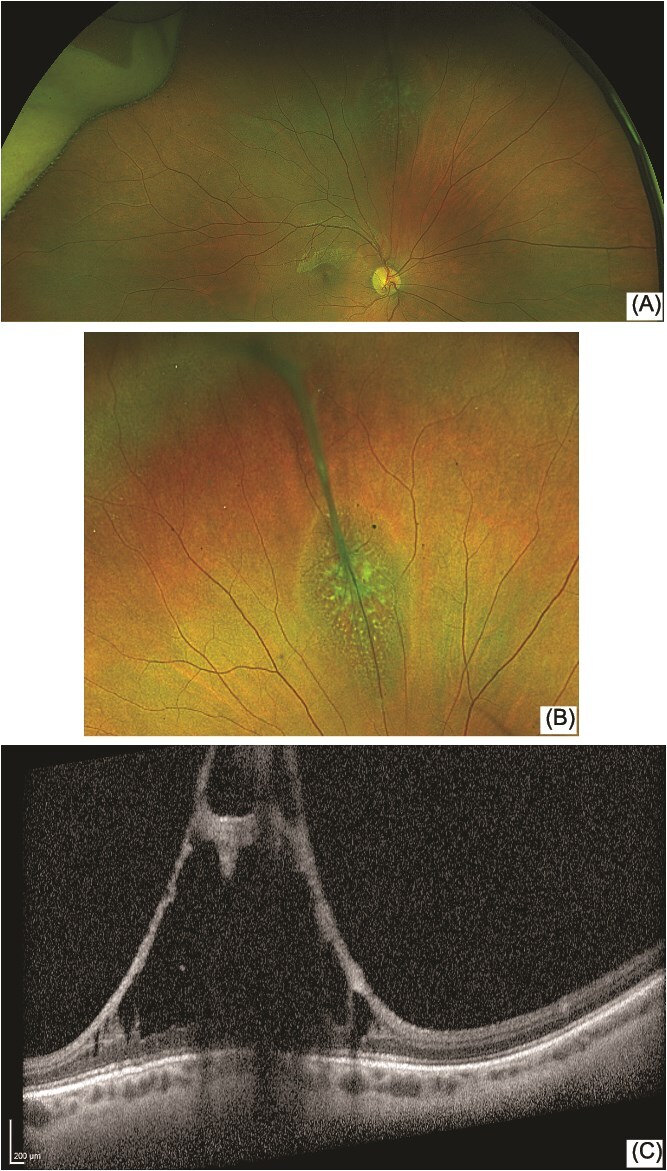
(A) and (B) Shows a wide-field image of the disc and superior retina. (C) Presents the OCT scan of the defect with the localised retinoschisis

Examination of the anterior segments showed no abnormalities. Posterior segment examination showed healthy optic. Figure 1 The right eye superior mid-peripheral retina showed a vessel branching off the superior branch of the retinal vein and extending anteriorly into the vitreous cavity while causing a localised retinal retinoschisis.

Features correspond to that of persistent foetal vasculature however, the vessel stalk emerges from the peripheral retina instead of the optic disc which is described in literature. The patient did not have any other systemic nor ophthalmic comorbidities expected with this condition [1–3].
